# Measurements of transepithelial electrical resistance (TEER) are affected by junctional length in immature epithelial monolayers

**DOI:** 10.1007/s00418-021-02026-4

**Published:** 2021-08-30

**Authors:** Kannapin Felix, Schmitz Tobias, Hansmann Jan, Schlegel Nicolas, Meir Michael

**Affiliations:** 1 Department of General, Visceral, Vascular and Pediatric Surgery, University Hospital Würzburg, Oberduerrbacherstrasse 6, 97080 Würzburg, Germany; 2 Department for Tissue Engineering and Regenerative Medicine, University Hospital Würzburg, Roentgenring 11, 97070 Würzburg, Germany; 3 Translational Center Regenerative Therapies (TLC-RT), Fraunhofer Institute for Silicate Research ISC, Neunerplatz 2, 97082 Würzburg, Germany

**Keywords:** TEER, Barrier models, Impedance spectroscopy, Permeability, Caco2 cells

## Abstract

The measurement of transepithelial electrical resistance (TEER) is a common technique to determine the barrier integrity of epithelial cell monolayers. However, it is remarkable that absolute TEER values of similar cell types cultured under comparable conditions show an immense heterogeneity. Based on previous observations, we hypothesized that the heterogeneity of absolute TEER measurements can not only be explained by maturation of junctional proteins but rather by dynamics in the absolute length of cell junctions within monolayers. Therefore, we analyzed TEER in epithelial cell monolayers of Caco2 cells during their differentiation, with special emphasis on both changes in the junctional complex and overall cell morphology within monolayers. We found that in epithelial Caco2 monolayers TEER increased until confluency, then decreased for some time, which was then followed by an additional increase during junctional differentiation. In contrast, permeability of macromolecules measured at different time points as 4 kDA fluorescein isothiocyanate (FITC)-dextran flux across monolayers steadily decreased during this time. Detailed analysis suggested that this observation could be explained by alterations of junctional length along the cell borders within monolayers during differentiation. In conclusion, these observations confirmed that changes in cell numbers and consecutive increase of junctional length have a critical impact on TEER values, especially at stages of early confluency when junctions are immature.

## Introduction

Measurements of transepithelial electrical resistance (TEER) are a standard technical approach to assess barrier properties and dynamics of various different in vitro cell culture models. TEER measurements are ideal for time-dependent and nondestructive monitoring of dynamic changes of barrier functions as shown in models of the endothelial barrier (Schlegel et al. 2012), intestinal epithelial barriers (Meir et al. 2019), pulmonary alveolar epithelial barrier (Rezaee and Georas 2014), or urinary tract epithelial barrier (Prot-Bertoye and Houillier 2020).

Historically, permeation or diffusion assays were a first approach to investigate the tightness of intercellular junctions (Bowman et al. 1983), where a tracer of defined molecular size was placed on the apical side of a cell monolayer that was grown on a semipermeable membrane. The flux of this tracer through the cell monolayer enabled the quantification of a permeability coefficient (Zelman 1972). Yet, most of the tracers themselves influence barrier properties, and the quantification is limited to certain time points since a continuous measurement is not possible (Duffy and Murphy 2001). The measurement of TEER offers the opportunity to monitor physiological homeostasis as well as pathological conditions in vitro in real time and in a noninvasive manner (Srinivasan et al. 2015).

In our previous work, we focused on mechanisms involved in the regulation of intestinal epithelial barrier function in vitro (Meir et al. 2015; Spindler et al. 2015). The intestinal epithelial barrier is maintained by junctional proteins forming the “terminal bar” that seals the paracellular cleft. This terminal bar is formed by circumferential intercellular proteins such as tight junctions or adherens junction proteins that are affected under several pathophysiological conditions (Schulzke et al. 2012; Ivanov and Naydenov 2013). In addition, desmosomes are critical in the regulation of the intestinal barrier (Schlegel et al. 2021).

Traditionally, for in vitro models of the intestinal epithelial barrier, redifferentiated cells from human colon carcinoma such as HT29/B-6 cells (Hering et al. 2017), T84 cells (Sayoc-Becerra et al. 2020), and Caco2 cells (Schlegel et al. 2010) have been used. However, when absolute TEER values expressed in Ω area were reported in these models, a wide variation of TEER values have been published in the last years even when similar experimental in vitro conditions for identical cell lines were applied. Different factors such as temperature (Blume et al. 2010), the passage number of cells (Briske-Anderson et al. 1997), or the composition of cell culture media (Robilliard et al. 2018) have been identified to influence TEER levels. While these factors can be easily controlled under laboratory conditions, we still observed a great variation of absolute TEER values in our own data especially during the early phase after confluency, in the differentiation phase of the cell monolayer. Based on our observations, we hypothesized that a major impact on absolute TEER measurements is the absolute circumferential length of cellular junctions within monolayers rather than the maturation of junctional proteins and the above-mentioned factors.

## Materials and methods

### Cell culture

Two different Caco2 cell lines were used for the experiments. Clone 1 was acquired from the Deutsche Sammlung für Mikroorganismen und Zelllkulturen (Braunschweig, Germany), while clone 2 was acquired from ATCC (Wesel, Germany). Both clones were cultured in Dulbecco’s modified medium supplemented with 50 U/ml penicillin G, 50 µg streptomycin, and 10% fetal calf serum (FCS) (Biochrom, Berlin, Germany) in a humidified atmosphere (95% air/5% CO2) at 37 °C.

### Immunocytochemistry

Immunostaining has been described in detail previously (Meir et al. 2020). In brief, epithelial cells were grown to confluence on coverslips and cultured to different time points as indicated below. Then, cells were fixated with 2% formaldehyde for 10 min at room temperature. After treatment with 0.1% Triton X‐100 for 15 min, monolayers were incubated at 4 °C overnight using the following primary antibodies (1:100 each in PBS): a mouse monoclonal E‐cadherin antibody IgG (# 610182, BD Biosciences, Heidelberg, Germany) and a mouse anti-Desmoglein2 IgG (# 32-6100, Thermo Fisher Scientific, Waltham, MA, USA), or mouse anti-Claudin1 IgG (# 37-4900 Thermo Fisher Scientific, Waltham MA, USA) and rabbit anti-Claudin5 IgG (# 34-1600 Thermo Fisher Scientific, Waltham MA, USA diluted 1:50 with PBS). As secondary antibodies, we used Cy3 (indocarbocyanine) labeled goat anti-mouse IgG (diluted 1:600; # 115-165-003, Dianova Hamburg Germany) or goat anti-rabbit IgG (diluted 1:600; # 111-165-003, Dianova, Hamburg, Germany). Coverslips and filters were mounted on glass slides with Vector Shield Mounting Medium as antifading compound, which included DAPI to counterstain cell nuclei (Vector Laboratories, Burlingham, CA). Representative experiments were photographed with a Keyence digital microscope (Keyence, Osaka, Japan) using a Zeiss lens (Carl-Zeiss Jena, Germany) with a 63× magnification and TRITC and GFP-BP as well as DAPI-BP filters (all Keyence, Osaka, Japan). Then, the images were taken with a 12.5 megapixel camera and analyzed with BZ-H4A Analysis Software (Keyence, Osaka, Japan).

### Measurements of cell number and circumferential junctional length

To quantify the number of cells and the circumferential junctional length per area during the differentiation, immunostaining with E-cadherin and DAPI was used. For each time point, the number of DAPI-stained nuclei in an area of 0.1 mm^2^ was counted. To estimate the circumferential junctional length, E-cadherin-stained cell borders were measured with ImageJ (National Institutes of Health, USA) and then divided by the number of cells in the same area of 0.1 mm^2^ to normalize the measurements.

### Measurement of FITC-dextran flux across monolayers of cultured epithelial cells

Caco2 cells were seeded on top of transwell filter chambers on 12-well plates (0.4 μm pore size; Falcon, Heidelberg, Germany) as previously described (Schlegel et al. 2010; Chang et al. 2000). After reaching confluence, cells were rinsed with PBS and incubated with fresh DMEM without phenol red (Sigma) containing 10 mg/ml FITC-dextran (4 kDa). Paracellular flux was assessed by taking 100 μl aliquots from the outer chamber over 2 h of incubation. Fluorescence was measured using a Tecan GENios Microplate Reader (MTX Lab systems, Bradenton, USA) with excitation and emission at 485 and 535 nm, respectively. For all experimental conditions, permeability coefficients (P_E_) were calculated by the following formula: ${\text{P}}_{{\text{E }}} = \frac{{\frac{{{\Delta C}_{{\text{A}}} }}{{{\Delta t}}} \times V_{A} }}{{S \times C_{L} }}$, where P_E_ = diffusive permeability (cm/s), ΔC_A_ = change of FITC-Dextran concentration, Δt = change of time, *V*_*A*_ = volume of the abluminal medium, S = surface area, and *C*_*L*_ = constant luminal concentration.

### Measurements of transepithelial electrical resistance (TEER)

ECIS 1600R for clone 1 (Applied BioPhysics) and ECIS ZTheta for clone 2 (Applied BioPhysics) were used to measure TEER of epithelial monolayers to assess epithelial barrier functions at 400 Hz, as described in detail previously (Flemming et al. 2015). The TEER of an empty insert was subtracted from every value. Medium was exchanged every 2 days.

### Statistics

Values are expressed as mean ± SD. Statistical analyses were performed using GraphPad Prism 7.0 (GraphPad, La Jolla, USA). Origin 2018 (OriginLab Corporation, Northampton, MA, USA) was used for creating graphs. For each experiment, Gaussian distribution was checked by D’Agostino and Pearson normality test. For every experiment, the statistic test used is stated in the figure legends. Statistical significance was assumed for *p* < 0.05.

## Results and discussion

### Caco2 cells need time to maturate to be considered as a true model of intestinal epithelial barrier function

**Fig. 1:**
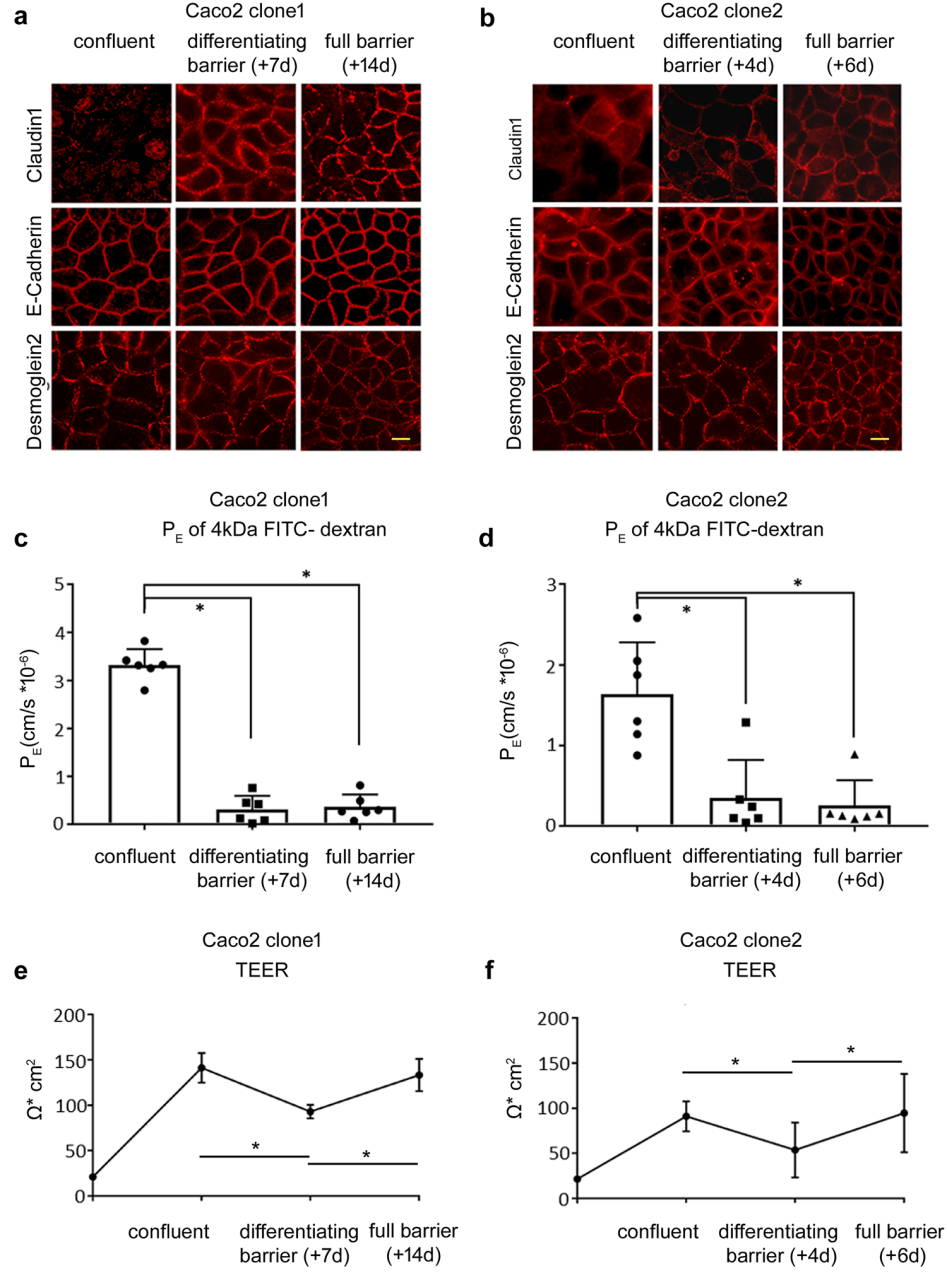
Differentiation of junctional properties of Caco2 cells. **a** Representative immunostainings during differentiation of the epithelial barrier of clone 1 are shown. At confluence, in contrast to E-cadherin, junctional proteins Desmoglein-2 (Dsg2) and Claudin-1 were not regularly located at the cell borders. This changed during differentiation of the monolayer to the state of full barrier 14 days later when all junctional proteins showed a regular staining at the cell borders (representatives are shown for *n* = 6, scale 20 µm). **b** Similar observations for the junctional proteins were seen for clone 2, where tight junction protein Claudin-1 was hardly detectable at the state of confluence but showed a linear staining at the cell borders at the moment of barrier differentiation after 4 days (representatives are shown for *n* = 6, scale 20 µm). **c**. Permeability of 4 kDa FITC-dextran revealed that, with the differentiation of junctional proteins of clone 1, the permeability coefficient (P_E_) declined to (0.31 ± 0.11) × 10^–6^ cm/s from (3.32 ± 0.13) × 10^–6^ cm/s at the state of confluence. This was not further altered under full barrier conditions 21 days after confluence where P_E_ was (0.36 ± 0.10) × 10^–6^ cm/s (*n* = 6, *p < 0.05, one-way ANOVA). **d**. The measurements of P_E_ for clone 2 paralleled the development of clone 1. At confluence, P_E_ was (1.64 ± 0.26) × 10^–6^ cm/s and dropped to (0.35 ± 0.19) × 10^–6^ cm/s 4 days post-confluence, which was not changed at full barrier properties after 6 days (*n* = 6, *p < 0.05, one-way ANOVA). **e**. Transepithelial electric resistance (TEER) of clone 1 during the differentiation of the cell monolayer is shown. After seeding, TEER was steadily increasing to 137 ± 16 Ω cm^2^. Then, over the next days, TEER decreased to 88 ± 7 Ω cm^2^ before it increased again to 139 ± 18 Ω cm^2^ until the state of full barrier was reached (*n* = 10, *p < 0.05, two-way ANOVA). **f**. This change of TEER over the time was almost identical in clone 2, where TEER rose to 87 ± 2 Ω cm^2^ at confluence, dropped during differentiation of the Caco2 cells, and then increased to 91 ± 1 Ω cm^2^ at full barrier (*n* = 10, *p < 0.05, two-way ANOVA)

First, we characterized the changes of the intestinal epithelial barrier function of two different Caco2 cell clones during their differentiation at different time points. To identify changes in the expression of junctional barrier proteins, we performed immunostaining. While at confluence both Caco2 clones showed regular staining patterns of adherens junction protein E-cadherin and desmosomal Desmoglein-2 at the cell borders, tight junction protein Claudin-1 was hardly visible (Fig. 1a, b). After 7 days, clone 1 demonstrated a maturated terminal bar at the cell borders where all junctional proteins including tight junctions were regularly distributed at the cell borders (Fig. 1a). Differentiated barrier maturation occurred earlier when analyzing clone 2, where a linear staining of tight junction proteins was already present 4 days after confluence (Fig. 1b). The staining pattern of all junctions did not change at later time points (14 days after confluence for clone 1, and 6 days after confluence for clone 2). Therefore, we considered the time point after 7 days for clone 1 and after 4 days for clone 2 as state of a differentiating barrier.

The differentiation of junctional proteins over time was paralleled by a continuous reduction in the permeability of 4 kDA FITC-dextran in the transwell model. This was revealed by measurements of permeability coefficient P_E_ at the day of confluence (confluent), after 7 days (clone 1) or 4 days (clone 2) (differentiating barrier), and 14 days (clone 1) or 6 days (clone 2) (full barrier) (Fig. 1c and d). The P_E_ at confluence was 3.32 ± 0.13 cm/s × 10^–6^ for clone 1 and 1.63 ± 0.26 cm/s × 10^–6^ for clone 2. P_E_ significantly decreased for both cell lines during maturation to 0.31 ± 0.11 cm/s × 10^–6^ for clone 1 and 0.34 ± 0.19 cm/s × 10^–6^ for clone 2, showing comparable P_E_ when the epithelial barrier was differentiated. After 14 days or 6 days, respectively, P_E_ was not further altered, indicating full barrier properties at these time points for both clones. In contrast to P_E_, measurements of TEER showed a different picture when barrier properties were assessed (Fig. 1e and f). Measurements were started directly after seeding of the cells. TEER of clone 1 increased to 137 ± 16 Ω cm^2^, when confluence was reached. Afterwards TEER dropped significantly to 88 ± 7 Ω cm^2^ and in the following increased again after 14 days to a final value of 139 ± 18 Ω cm^2^. A comparable course of TEER development was observed for clone 2, where TEER initially increased to 87 ± 2 Ω cm^2^ and dropped to 49 ± 4 Ω cm^2^ after 4 days until TEER again increased to 91 ± 1 Ω cm^2^ after day 6. In summary, despite a continuous stabilization of epithelial barrier function as revealed by measurements of 4 kDa FITC-dextran flux, TEER values under the same conditions are undulating. It is important to note that, although the assessment of the barrier function can be performed by measuring the TEER value as well as by studying the passage of marker molecules, the two experimental approaches depend on different transport mechanisms through the barrier (Zucco et al. 2005). This means that, depending on the status of the cell monolayer, the two techniques may express different levels of observed barrier tightness. While the measurement of permeability of FITC-dextran or other non-electrolyte paracellular tracers through the cell monolayer depends on the paracellular water flow and the pore size of the tight junctions, the measurement of the TEER value depends on the ionic conductance of the paracellular pathway (Zucco et al. 2005).

### Junctional length of Caco2 cells increased during maturation

**Fig. 2:**
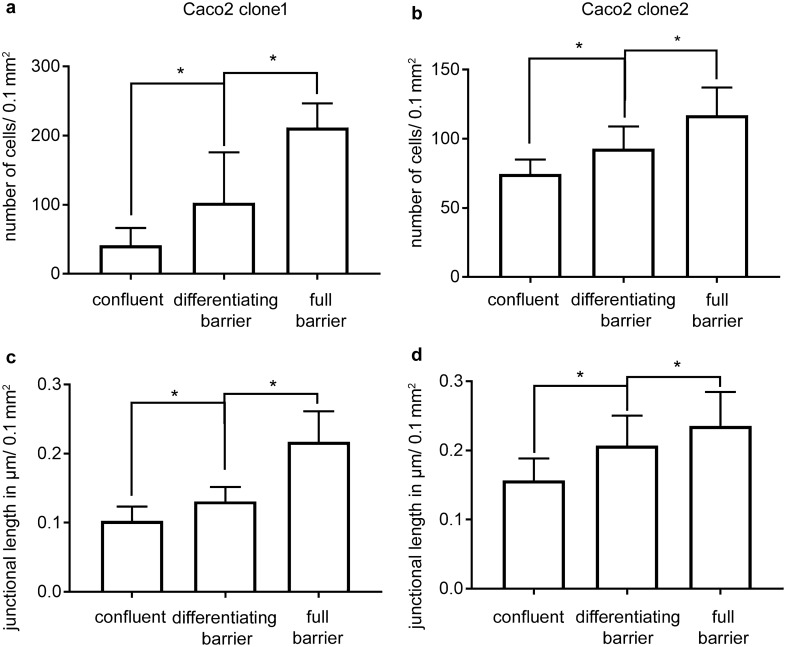
Changes in cell numbers and junctional length during differentiation. **a** The changes in the number of cells during the differentiation of clone 1 are shown. The number of cells/0.1 mm^2^ steadily increased until day 14, where a maximum of 211 ± 3 per 0.1 mm^2^ was reached (*n* = 8, *p < 0.05, one-way ANOVA). **b**Likewise, the average number of cells per 0.1 mm^2^ of clone 2 rose through the different states of differentiation (*n* = 8, *p < 0.05, one-way ANOVA). **c**. The increase in cell number was paralleled by an increase of the junctional length/µm. The junctional length of clone 1 increased from 0.10 ± 0.03 µm/0.1 mm^2^ to 0.22 ± 0.04 µm/0.1 mm^2^ at the state of full barrier (*n* = 8, *p < 0.05, one-way ANOVA). **d**. Similarly, in clone 2 the junctional length expanded over time (*n* = 8, *p < 0.05, one-way ANOVA)

Although cells were confluent, we still noticed an increase in the number of cells and a size reduction of cells during the maturation of Caco2 monolayers. This was confirmed by counting the number of cells/area at the different time points: for clone 1, number of cells/0.1 mm^2^ increased from 41 ± 2 at confluence to 102 ± 7 at day 7 to 211 ± 3 at day 14 (Fig. 2a). A comparable increase was observed for clone 2, where number of cells/0.1 mm^2^ augmented from 74 ± 2 at confluence to 93 ± 3 at day 4 to 117 ± 3 at day 6 (Fig. 2b). Given that the absolute contact area between cells changed during cell growth after confluency, we hypothesized that the undulation of TEER after confluence could be explained not only by the maturation of junctional proteins but also by an increase of absolute circumferential junctional length during the differentiation of the enterocytes. The increase of circumferential junctional length was confirmed since average µm junctional length/0.1 mm^2^ for clone 1 increased from 0.10 ± 0.02 µm/0.1 mm^2^ to 0.22 ± 0.04 µm/0.1 mm^2^ (Fig. 2c) after 14 days, and from 0.16 ± 0.03 µm/0.1 mm^2^ to 0.24 ± 0.05 µm/0.1 mm^2^ for clone 2 (Fig. 2e).

### Changes in TEER were reciprocal to changes in junctional length or cell numbers only in the early stage after confluence

**Fig. 3:**
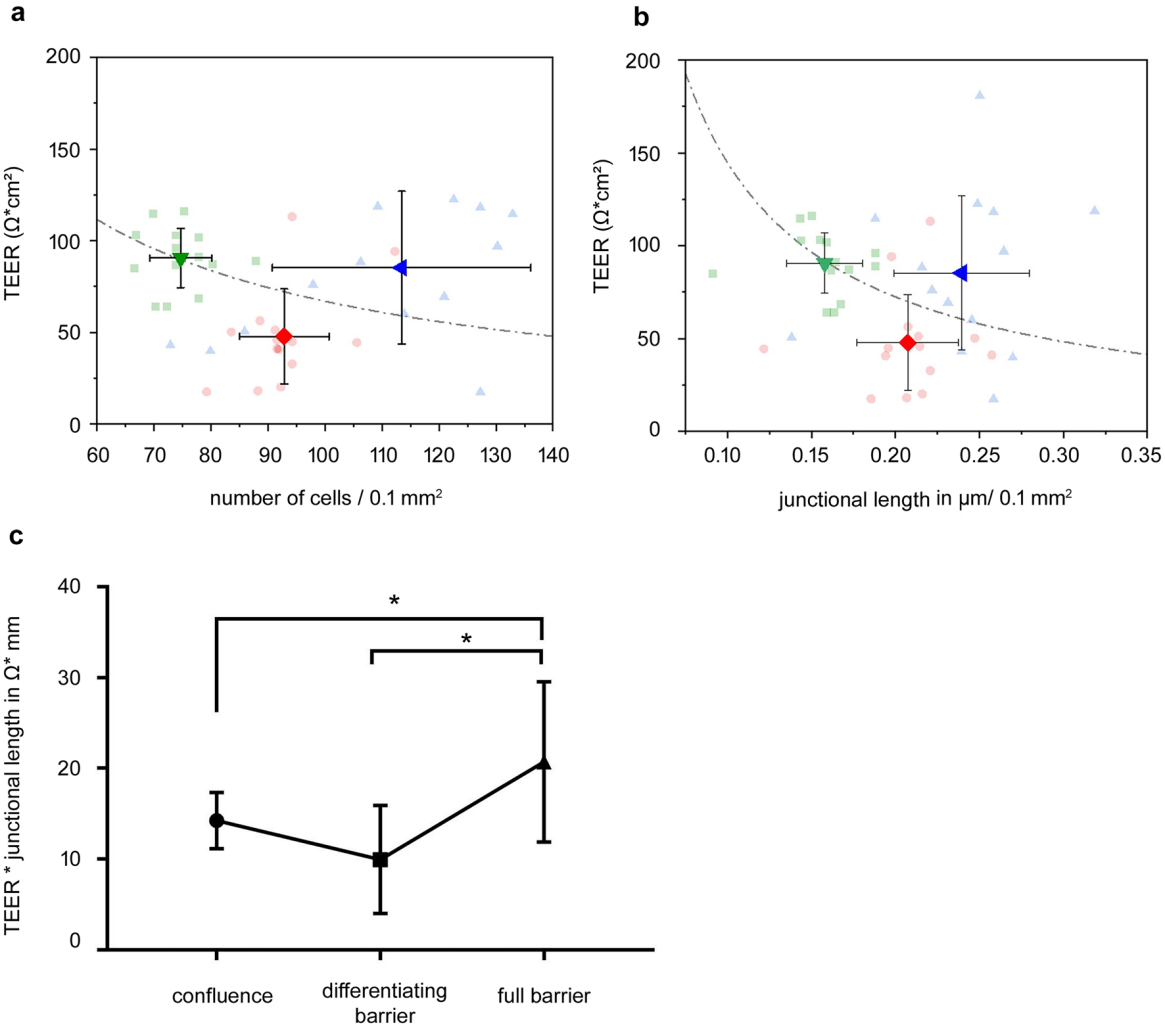
Changes of TEER and corresponding cell numbers and junctional length. **a** A plot of the individual transepithelial electric resistance (TEER) of clone 2 to the corresponding number of cells is shown. This demonstrated that, from confluence to the state of differentiating barrier, the values dropped reciprocal to the increase in number of cells, then increased to the level of full barrier properties, though the number of cells was even higher than in previous states. If the TEER solely depended on cell numbers at the state of full barrier, we would have expected values that followed the ideal logarithmic curve. **b** Similar effects were observed when we drafted TEER to the corresponding measurements of clone 2 of junctional length in µm/0.1 mm^2^, where the increase of TEER under full barrier properties also did not follow a reciprocal curve. **c**. To differentiate if the TEER per cell–cell junction would remain constant, we multiplied the TEER with the corresponding junctional length of the same experiment. This revealed that, during the initial differentiation from confluence to differentiating barrier, the TEER per junctional length stayed nearly constant from 14.2 ± 3.1 Ω mm to 10.0 ± 5.9 Ω mm, while a significant increase to 20.7 ± 8.8 Ω mm was observed under full barrier conditions (*n* = 8, *p < 0.05, two-way ANOVA)

To elucidate whether the changes in cell numbers and junctional length would be the critical event that explained our observation of undulating TEER values after confluence, we plotted the TEER values from the Caco2 clone 2 cell culture models against the average number of cells counted and the circumferential junctional length in an area 0.1 mm^2^ (Fig. 3a, b). This graphical representation provided a clearer view of how the growing number of cells and the junctional length correlated with the measured TEER values. Following the hypothesis of a resistor with a growing surface area, the increasing junctional length should then lead to reciprocal changes of the TEER. This would be only the case if the resistance per length of the cell–cell junctions remained constant. To address this, we multiplied the individual TEER values in Ω cm^2^ with the corresponding junctional length in µm/0.1 mm^2^ of the same experiment. This revealed that, this calculated value from state of confluence (14.2 ± 3.1 Ω mm) to the differentiating barrier (10.0 ± 5.9 Ω mm) did not show an significant changes. However, it increased to 20.7 ± 8.8 Ω mm under full barrier conditions, indicating that during that phase the paracellular resistance per junctional length changed dramatically. Therefore, our calculations indicated that the junctional length/area directly affects TEER measurements only at the very early stage of junctional maturation, when tight junctions are not yet functional. This explains our transitional drop after cells were confluent. However, when junctional differentiation starts, the dynamics of the TEER rather reflect barrier maturation, whereas junctional length becomes negligible.

## Summary and conclusion

**Fig. 4:**
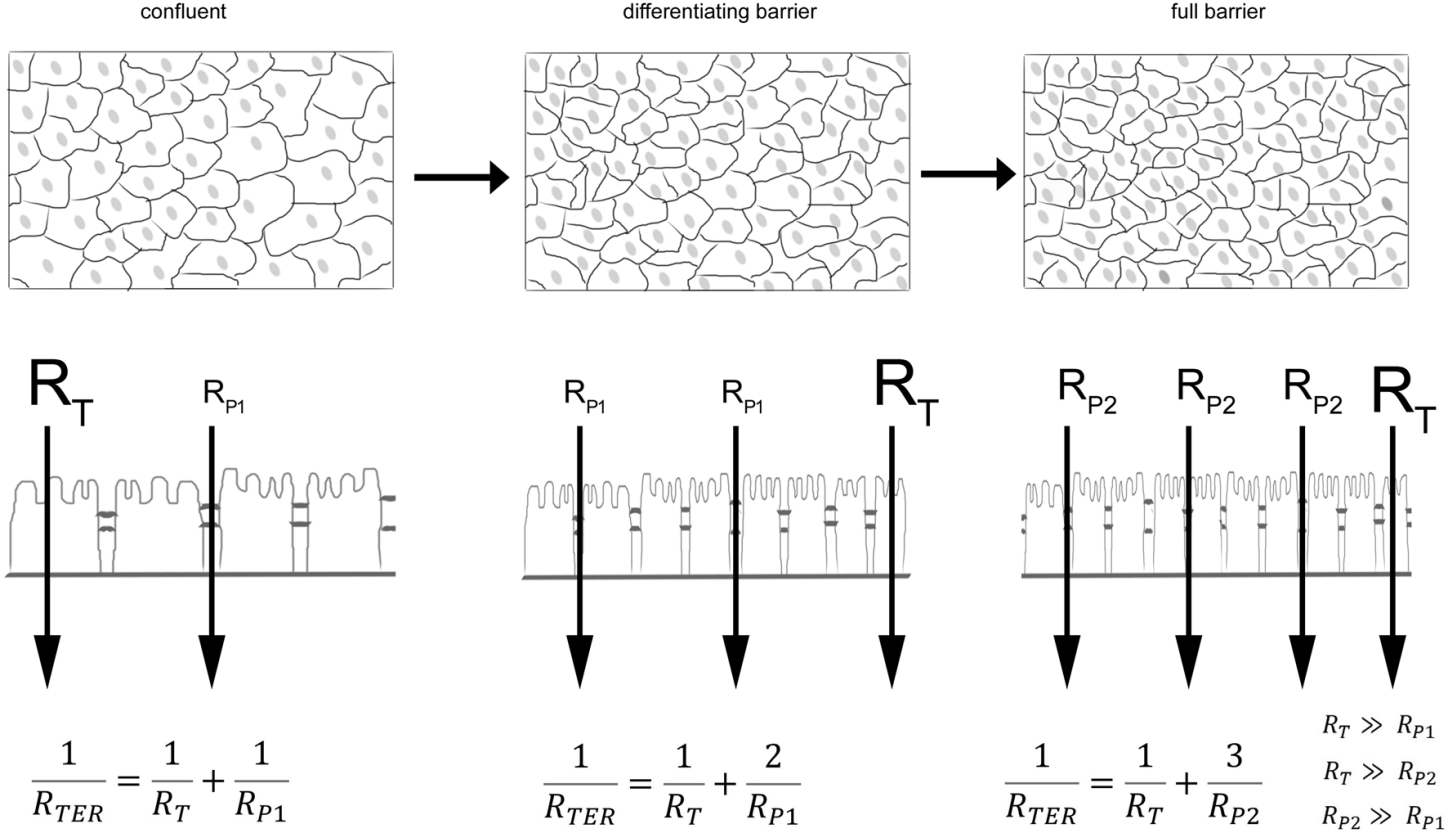
Schematic synopsis of effect of junctional length and TEER. A schematic synopsis of the observations is shown. At the state of confluence, the overall TEER was determined by the lower resistor for the paracellular pathway (R_P1_) compared with the resistor of the transcellular pathway (R_T_). During differentiation, an increase in number of cells and junctional length influenced the overall TEER until the paracellular resistor R_P2_ sufficiently increased when full barrier properties were achieved. The effect is clearer if you assume that TEER is following a parallel circuit. The equation to determine the TEER in such a parallel circuit clearly demonstrates that an increase in junctional length by the factor 2 leads to a reduction of TEER by 50% if all factors determining the resistor R_P1_ remain constant and R_T_ is distinctly larger than R_p_. This effect is diminished if R_P2_ is larger than the initial R_P1_

Taken together, we observed that during the differentiation of a confluent cell monolayer the TEER initially decreased, while permeability of 4 kDa FITC-dextran and immunocytochemistry of cellular junctions would suggest a steady increase of TEER. Our data clearly show that the growing number of cells and, thus, junctional length dominate the TEER values in the early stages of the maturation process (confluent–differentiating barrier). However, in the later stages of this process (differentiating barrier–full barrier), the strength of the intercellular connections is increasing, and despite rapidly increasing cell numbers and junctional length, a continuous strengthening of the barrier can be observed, resulting in an increase in paracellular resistance and, thus, rising TEER values (Fig. 4). This is important when assessing barrier maturation by TEER since confluent monolayers may exhibit dynamics that may lead to misinterpretations after having reached confluence. According to our observation reported here, this depends on strong alterations of overall junctional length within monolayers. Therefore, we strongly recommend thorough assessment of morphology and maturation state of junctions in parallel when TEER measurements are applied for barrier measurements.

## Data Availability

All data generated or analyzed during this study are included in this published article.
